# Volatile Organic Compounds Profiles to Determine Authenticity of Sweet Orange Juice Using Head Space Gas Chromatography Coupled with Multivariate Analysis

**DOI:** 10.3390/foods9040505

**Published:** 2020-04-16

**Authors:** Qi Zhou, Guijie Li, Zhu Ou-Yang, Xin Yi, Linhua Huang, Hua Wang

**Affiliations:** 1 Citrus Research Institute, Southwest University, Chongqing 400712, China; zhouqi1834@163.com (Q.Z.); liguijie@cric.cn (G.L.); 15213108545@163.com (Z.O.-Y.); yixin950501@163.com (X.Y.); huanglh@cric.cn (L.H.); 2 National Citrus Engineering Research Center, Chinese Academy of Agricultural Sciences, Chongqing 400712, China; 3 Chongqing Collaborative Innovation Center for Functional Food, Chongqing University of Education, Chongqing 400067, China

**Keywords:** mandarin juice, not from concentrate (NFC), principal component analysis (PCA), headspace solid-phase microextraction (HS-SPME)

## Abstract

An efficient and practical method for identifying mandarin juice over-blended into not from concentrate (NFC) orange juice was established. Juices were extracted from different cultivars of sweet orange and mandarin fruits. After being pasteurized, the volatile organic compounds (VOCs) in the juice samples were extracted using headspace solid-phase microextraction, and qualitatively and quantitatively analyzed using gas chromatography–mass spectrometry detection. Thirty-two VOCs contained in both the sweet orange juice and mandarin juice were used as variables, and the identification model for discriminating between the two varieties of juice was established by principal component analysis. Validation was applied by using common mandarin juices from Ponkan, Satsuma and Nanfengmiju cultivars blended at series of proportions into orange juices from Long-leaf, Olinda, and Hamlin cultivars. The model can visually identify a blending of mandarin juice at the volume fraction of 10% or above.

## 1. Introduction

Orange juice is one of the most popular fruit juices because of its high vitamin C content, rich flavor, and balanced sweetness and sourness. According to the United Nations Commodity Trade Database, in 2017, the global export volume and value of citrus juices reached 5.853 million tons and 6769 million US dollars, respectively, in which orange juice accounted for more than 85% of the total amount. Among all common juice products, not from concentrate (NFC) orange juice has been considered as being of the best quality, accounting for an important share of orange juice consumption in developed countries, and its share in emerging countries’ markets has also increased year by year [1,2]. Orange juice is generally allowed to be mixed with a small amount of other citrus juice or juice cells, according to Codex 247-2005 of the Codex Alimentarius Commission, to adjust its taste and flavor before sterilized packaging. However, excessive addition will reduce the quality of orange juice and affect the sensory experience of consumers (data from FAO, 2019) [3].

The authenticity of NFC orange juice is a prerequisite to ensure its quality. With the rapid development of the international juice industry, European fruit juice association (AIJN) and the international juice industrial protection association (SGF) have set standards for authenticity testing of fruit and vegetable juices. However, the analysis of detection methods is complex and time-consuming. Other methods for identifying the quality of orange juice have been developed [4,5,6,7,8,9]. Bocharova et al. compared the odor intensity in commercial and fresh-squeezed orange juice by GC–MS and dilution. It was found that the odor intensity of commercial orange juice with added artificial flavoring was two times higher than that of fresh-squeezed orange juice, and the odor intensity of commercial orange juice without artificial flavoring was 1.5–2.5 times lower than that of fresh-squeezed orange juice. The commercial orange juice was made by diluting the orange juice concentrate, which had certain reference significance for authenticity identification of orange juice [10]. Cuevas et al. used high performance liquid chromatography–high resolution mass spectrometry (HPLC–HRMS) and headspace solid-phase microextraction–gas chromatography–mass spectrometry (HS-SPME–GC–MS) to analyze the metabolomics fingerprint and volatile components, such as flavonoids, fatty acids, aldehydes, and esters of commercial orange juice, established an optimal category model, and preliminarily achieved the identification of organic orange juice and conventional orange juice [11]. Shen et al. used electronic nose technology and Fourier transform attenuated total reflection infrared spectroscopy (ATR-FTIR) combined with principal component analysis (PCA) and linear discriminant analysis (LDA), and the results showed that there were significant differences in the flavor between freshly squeezed juice and that of concentrated juice [12]. In addition, nuclear magnetic resonance [13,14] and molecular imprinting [15] were used to identify the quality of orange juice recently. Among these studies in orange juice quality and authenticity detection, many were based on establishing a database of certain chemical characteristics of orange juice [16,17,18,19]; however, due to the very limited number of samples adopted in most studies [20,21,22], the established databases could hardly be applied to actual detection of juice fraudulence. 

The principal component analysis is a matrix compression algorithm that reduces the dimensionality of original variables with a certain correlation and combines them into a new set of unrelated comprehensive variables, to replace the original variables but retain the information of the original matrix as much as possible. This makes it easier for researchers to discover features and correlations between sample variables from large amounts of data. In the field of food analysis, where there are usually large numbers of indicators of the samples to be measured, PCA is often used to conduct dimensionality reduction treatment on the data, and key comprehensive indicators are selected to reflect the original information of the samples [23,24].

Volatile organic compounds (VOCs) in citrus juice are a class of secondary metabolites produced. It has been found that sweet orange juice and mandarin juice have different VOCs [25,26,27], and the difference between them can be used to identify whether orange juice contains excessive mandarin juice. Given this, we have collected a full range of varieties of citrus that can be used for juicing and prepared them into NFC citrus juice. Then, by analyzing the distribution characteristics and differences of VOCs in sweet orange juice and mandarin juice, a recognition model of sweet orange juice and mandarin juice can be established by using PCA. Sweet orange juice mixed with a series of percentages of mandarin juice can be identified by this model [28]. 

## 2. Materials and Methods 

### 2.1. Citrus Materials and Sample Preparation

Mature sweet orange fruit and mandarin fruit were collected from orchards in Jiangjin and Zhongxian, Chongqing, China and the orchards of the National Citrus Germplasm Repository in Xiema, Beibei District, Chongqing, China (106°43′ E 29°83′ N), or purchased from local supermarkets. The samples included 27 sweet orange cultivars and 19 mandarin cultivars. 

Preparation of NFC juice: Fresh and mature citrus fruits were manually peeled, and juices were extracted using a Hurom model H-100-DWBIA0 rotary juice extractor, respectively. The juice was filtered through 100-mesh gauze and collected in a food-grade stainless steel pot. The pot was heated by immersion in a thermostatic water bath at 65 °C and stirred at 240 rmp. The juice was heated for 30 min to mimic thermal processing conditions used in the juice industry. After heating, the juice was bottled and immediately put into an ice bath to quickly cool to 4–5 °C. Total soluble solids of the NFC juices were determined by using a Pal-1 handheld Brix meter (ATAGO, Tokyo, Japan). 

Preparation of mixed juices for authenticity test: Sweet oranges and mandarins commonly used for juice were selected and extracted, and their juices were mixed in different volume proportions (v:v = 5:95, 10:90, 15:85, 20:80, 25:75, and 30:70). Three groups of mixed juice, Olinda Valencia orange and Xinshengxi No.3 Ponkan, Hamlin orange and 2003-4 Satsuma, and Long-leaf orange and Nanfeng mandarin, were prepared to test the differentiation ability of the discriminant model.

### 2.2. Standards and Agents

Cyclohexanone (>99.5%, internal standard) was purchased from Aladdin Industrial Co. (Shanghai, China), and diluted 40 times by methanol. C5-C25 N-alkanes and methanol were purchased from Honeywell Co. (New Jersey, USA). Fructose, glucose, sucrose, citric acid, and vitamin C were purchased from Solarbio Science & Technology Co. (Beijing, China).

### 2.3. Analysis of VOCs by HS-SPME–GC–MS

Five milliliters of shaken fruit juice was put into a 20 mL glass vial. Three microlitres of the internal standard were accurately added into the glass vial, and the headspace was filled with nitrogen. The sample was equilibrated for 20 min at 40 °C in water. Solid-phase microextraction was performed by exposing a divinylbenzene/carboxen/polydimethylsiloxane (DVB/CAR/PDMS; Supelco, Bellefonte, PA, USA) StableFlex fiber (1 cm, 50/30 μm) in the vial headspace of the sample at a constant depth at 40 °C for 30 min. The samples were gently vortexed during equilibration and extraction at 240 rpm, using a magnetic stirrer. After extraction, the fiber was removed from the vial and immediately inserted into the injection port of the GC for desorption at 250 °C for 5 min. All analyses of the volatile compounds were performed in triplicate.

The gas chromatography analyses were carried out using Agilent-7890B gas chromatography (GC) equipped with a mass selective detector (MSD). Volatile compounds were separated on an Agilent DB-5 MS column (30 m × 0.25 mm, 0.25 μm film thickness), with helium as the carrier gas at a constant flow of 1.2 mL/min. The oven temperature conditions were 35 °C for 0 min, increasing at 7 °C min^−1^ to 98 °C, 3 °C min^−1^ to 161 °C, 10 °C min^−1^ to 241 °C, and then held for 16 min. Electronic ionization was used (70 eV); the ion source and transfer line temperatures were 230 °C and 280 °C, respectively. Detection was performed in scan mode in the range between 40 and 350 m/z.

### 2.4. Determination of Detection Limit and Quantitative Limit

Model mandarin juices were prepared using the formulation used by PELEG’s method [29] with slight modification. The basic model juice contained fructose (3.0 g), glucose (3.0 g), sucrose (6.0 g), and citric acid (1.0 g), dissolved using Milli-Q water and diluted to a final volume of 100 mL. Vitamin C was added to the simulated juice at a concentration of 0.3 g/L. Using model mandarin juices as a blank sample and cyclohexanone as an internal standard, the determination of extremely small amounts of VOCs was carried out following the requirements of the International Council for Harmonization (ICH) for the Registration of Technical Requirements for Human Drugs and the Chinese Pharmacopoeia. Different concentrations of internal standard samples were tested, and the noise ratio calculation formula was used to calculate the detection limit (pk-pk S/N = 3) and the quantitative limit (pk-pk S/N = 10) of this experimental method. The detection limit was 9.28 × 10^−5^ μg/mL, and the limit of quantification was 2.78 × 10^−4^ μg/mL, which ensured that the VOCs needed for identification could be detected and quantified. 

### 2.5. Identification of Volatile Organic Compounds

The mass spectrometry and retention index of the compound were matched and compared with the Agilent W10N14.l mass spectrometry library and retention index database, respectively. The retention index database was provided by the University of Florida (http://www.flavornet.org/flavornet.html) [30]. The retention index of the mixture was calculated with N-alkane standard (C5–C20) under the same conditions as those of the sample, and the difference between the calculated retention index and the database retention index was less than 5%, which ensured the accuracy of the results.

Quantitation was performed according to Shui et al.’s quantitative method [31]. The internal standard method was used, and the internal standard was cyclohexanone (14.25 µg/L). The semi-quantitative analysis was performed by comparing the peak area of each component with that of the internal standard, and the content unit was μg/mL. Samples of each variety were prepared three times and injected, respectively. All samples used the same preparation method. The results are expressed as the mean of three samples. 

### 2.6. Statistical Analysis

Chromatograms and spectra were recorded and processed using Masshunter Qualitative Workflows B.08.00 software. The data analysis software Unscrambler X 10.4 was implemented. Data processing was performed using Microsoft Excel 365. Image processing was performed with Origin 2018.

The principal idea of principal component analysis is to reduce a quantity set that includes many interconnected variables and keep as many useful variables as possible in the quantity set. In this study, we used PCA to recombine VOCs that needed to be analyzed in sweet orange juice and mandarin juice, forming a new comprehensive index that reflected the characteristics of the sample.

PCA analysis steps: The category of VOCs was taken as the row of the matrix, and the sample to be tested was taken as the column of the matrix. Data were inserted, and principal component analysis was performed. A maximum of four components was selected for principal component analysis.

## 3. Results and Discussion

### 3.1. VOCs Profiles of Different Varieties of Sweet Orange Juice and Mandarin Juice

#### 3.1.1. Distribution Characteristics of VOCs in Sweet Orange and Mandarin Juices

**Figure 1:**
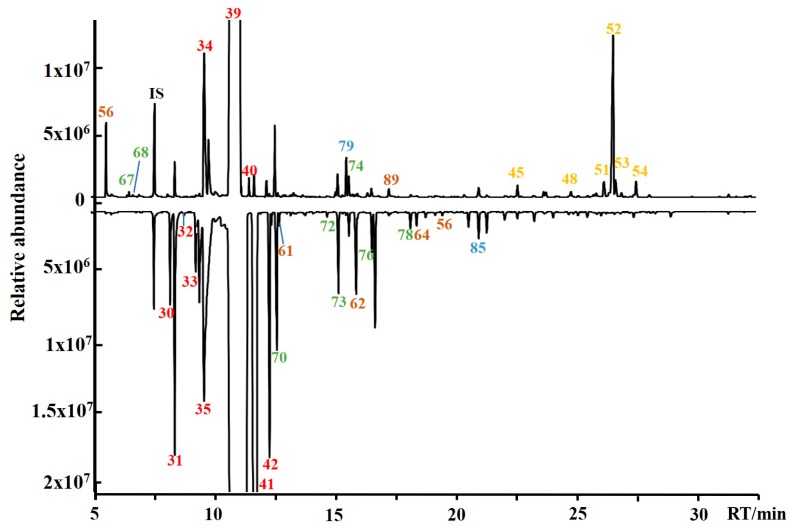
Total ion flow chromatogram of a typical sweet orange juice (above: Olinda Valencia orange juice) and a mandarin juice (below: Xinshengxi No.3 Ponkan juice). Compound numbers correspond to those in Table 1 and Table 2. Each type of substance is shown in different colors: red: monoterpenes; yellow: sesquiterpenes; brown: aldehydes and ketones; green: alcohols; and blue: esters. IS: internal standard.

All juices had a Brix of 10.5–13.5 which ensured their acceptability for juicing. Detailed information on each juice sample is shown in Table S1. Volatile organic compounds in 27 sweet orange and 19 mandarin heated juices were determined, and their typical total ion chromatograms (TIC) are shown in Figure 1 (above is Olinda Valencia orange juice, and below is Xinshengxi No.3 Ponkan juice). Orange and mandarin juice TICs were mirrored for a clear comparison. Among all VOCs, D-limonene (39) had the highest concentration. The VOCs of both varieties of juice were composed of monoterpenes, sesquiterpenes, aldehydes, esters, alcohols, and ketones. The chromatographic elution can be roughly divided into four sections. In Section 1, low carbon alcohols and aldehydes flowed out in 5.0–8.0 min. In Section 2, monoterpenes flowed out in 8.0–12.5 min. In Section 3, alcohols, aldehydes and esters with higher molecular weight flowed out in 12.5–22.0 min. In Section 4, sesquiterpenoids flowed out in 22.0–32.0 min. It can be seen from the Figure 1. that there are differences in the types and concentrations of VOCs in sweet orange juice and mandarin juice. This is also consistent with the existing research results [28].

#### 3.1.2. Characteristic VOCs in Sweet Orange Juice and Mandarin Juices

After comparison, the characteristic VOCs, which were exclusively contained in NFC sweet orange juice or NFC mandarin juice, respectively, are shown in Table 1. According to the table, among the 27 varieties of sweet orange juices and 19 varieties of mandarin juices, there were 20 VOCs unique to sweet orange juice, including four sesquiterpenoids, three aldehydes, four alcohols, six esters, and three ketones. There were also nine VOCs unique to mandarin juice, including four sesquiterpenoids, one aldehyde, two alcohols, and two ketones. Neither of the characteristic volatile organic compounds in sweet orange juice or mandarin juice contained monoterpenoids. Esters have been well-known to be a major aroma contributor to citrus juice [32]. However, in the characteristic VOCs, sweet orange juice contained six esters, which were not contained in the mandarin juice. At the same time, the aroma threshold of volatile ester was lower. This may be one reason why sweet orange juice has a better overall aroma than mandarin juice.

The characteristic VOCs of mandarin juice can be used to determine if mandarin juice is added to the orange juice. Orange juice samples that contained mandarin-specific VOCs could be preliminarily determined as not 100% orange juice. The sample juice was then further evaluated for how much mandarin juice was blended. 

#### 3.1.3. Common VOCs in Both Sweet Orange Juice and Mandarin Juices

By further analysis, the common VOCs in sweet orange juice and mandarin juice are shown in Table 2. In both the sweet orange juices and mandarin juices, terpenes were the most common, including 15 monoterpenes and 11 sesquiterpenes; the number of alcohols, aldehydes, and esters were all approximately 10, and ketones were the least, with three detected. A total of 61 volatile substances were shared in both groups, proving that sweet orange juice and mandarin juice have most of the same VOCs constituent. 

By eliminating D-limonene, which was contained in almost all citrus species with a high concentration and the components with a content of 0 or below the quantitation limit, 32 common VOCs of sweet orange juice and mandarin juice were selected and shown in Tables S2–S5. Concentrations of the selected common monoterpenes in each juice samples are shown in Table S2. The contents of β-pinene (34), α-terpinene (38), and γ-terpinene (41) in mandarin juice were significantly higher than those in sweet orange juice (*p* < 0.005). β-trans-ocimene (40) and L-carvone (89) in sweet orange juice were significantly higher than that in mandarin juice (*p* < 0.005). However, the overall concentrations of common monoterpenes in mandarin juice were significantly higher than those in sweet orange juice, and the content of β-myrcene was higher than that of other monoterpenes in both sweet orange juice and mandarin juice. According to Table S3, the content of β-selinene (51), valencene (52), and α-selinene (53) in sweet orange juice was significantly higher than that in mandarin juice (*p* < 0.005). As most sweet orange juice does not contain γ-muurolene (49), this may be related to the variety and cultivation environment. Also, the overall concentrations of common sesquiterpenes in sweet orange juice were significantly higher than those in mandarin juice. However, linalool (70) was one of the most abundant alcohols in both citrus juices. This is also consistent with the results of previous studies [28]. In addition, the total amounts of common alcohols, aldehydes, and esters in sweet orange and mandarin juices showed no significant difference in Tables S4–S5. It can be seen that the main differences in the concentration of common VOCs in sweet orange juice and mandarin juice exist in terpenoids. These characteristics in Tables S2–S3 also coincide with the distribution characteristics of these common VOCs in Figure 1, and can help to establish the identification model.

### 3.2. Principal Component Analysis of VOCs in Samples

**Figure 2:**
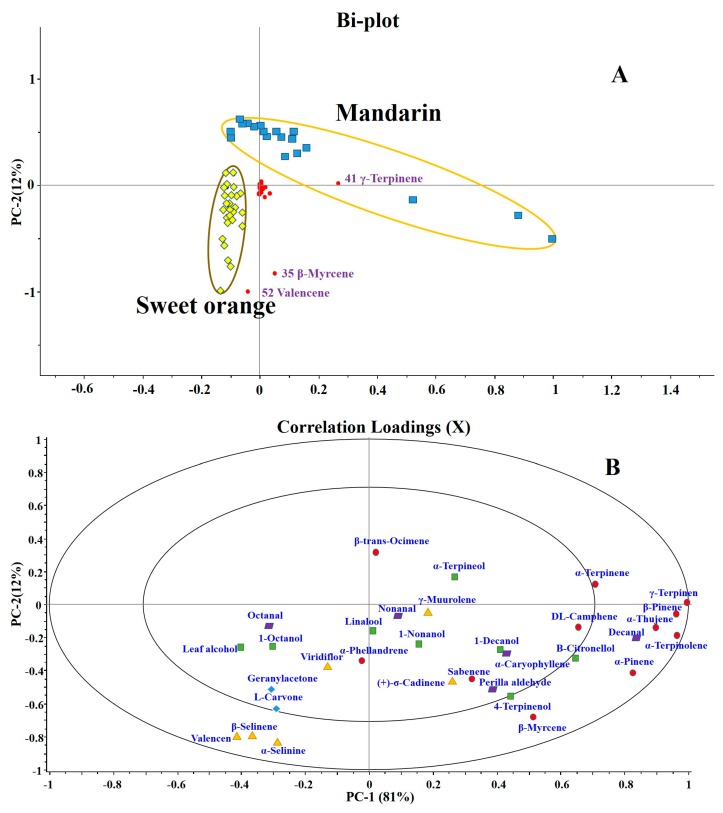
Principal component analysis of sweet orange juices and mandarin juices. (**A**) Scores and loadings biplot. Sweet orange juice and mandarin juice sample scatters are marked with different shapes and grouped; the red dots represent variables whose position distribution indicates their contribution to the sample scores. (**B**) Correlation loadings of each variable. Each type of substance is shown in different shapes and colors.

To further elucidate the difference of common VOCs between sweet orange and mandarin juice, all collected citrus juices were analyzed using principal component analysis. The 32 common volatile components were used as variables, and the results are shown in Figure 2. As seen from the two-dimensional biplot (Figure 2A), the cluster of sweet orange juice samples was well separated from that of mandarin juice samples. The red dots represent variables whose position distribution indicates their contribution to the sample scores, in which γ-terpinene (41), β-myrcene (35), and valencene (52) had more significant contributions to samples. Figure 2B is a correlation loading plot, which provides a scale independent assessment of the VOCs and a clearer indication of variable correlations. In Figure 2B, the contribution of γ-terpinene (41), α-thujene (30), β-pinene (34), α-terpinolene (42), decanal (62), and α-pinene (31) to PC-1 reached between 0.8 and 1.0, while that of valencene (52), α-selinene (53), β-selinene (51), and β-myrcene (35) to PC-2 reached 0.8−0.9. Among these, most of the major contributions in the X+ direction were monoterpenoids, and β-myrcene (35) contributed to both the X+ and Y- directions, which means that it made a great contribution to clusters of sweet orange and mandarin. In addition, the substances that contributed significantly to the X- and Y- directions were sesquiterpenes (valencene (52), α-selinene (53), and β-selinene (51)). Combining with Figure 2A,B, valencene (52) and γ-terpinene (41) made the largest contribution to clusters of sweet orange juice and mandarin juice, respectively. These VOCs play a key role in differentiating between sweet oranges and mandarin oranges. At the same time, this also shows that the spatial relationship between the scores and loadings biplot and the correlation loading plot correspond with each other. 

**Figure 3:**
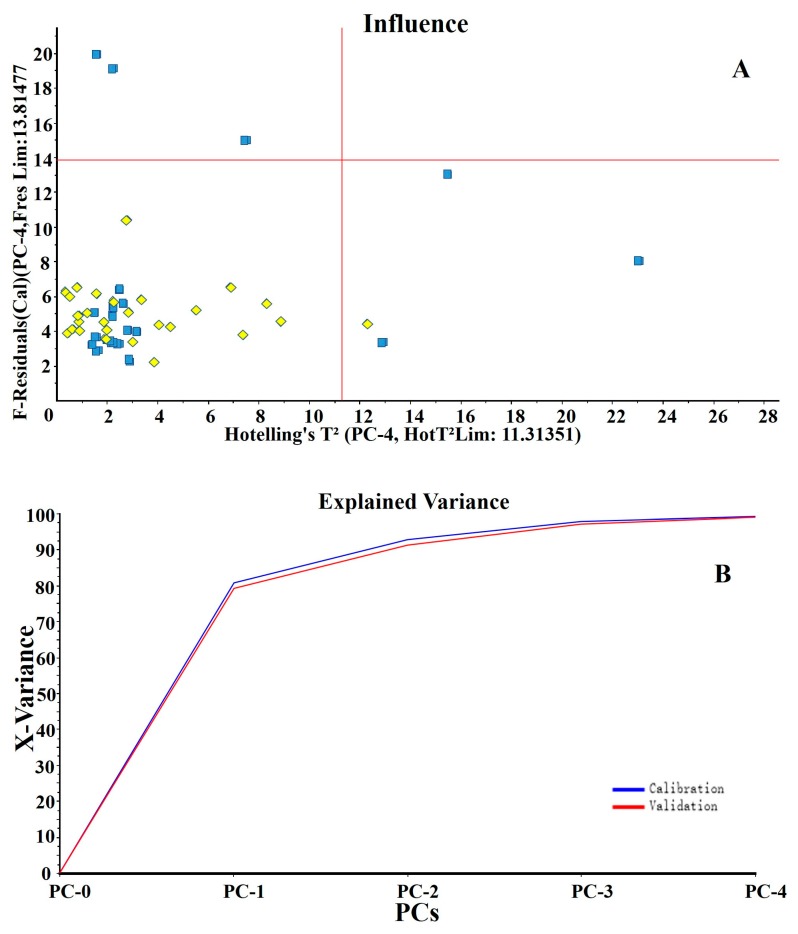
Hotelling’s T test results and cumulative interpretable variance of the principal components of orange juice samples. (**A**) Influence plotting. The sweet orange juices are marked in yellow diamond dots and mandarin juices are marked in blue squares. (**B**) The explained variance. The calibrated and validated curves are compared.

The above results show that the distribution of VOCs in sweet orange juice and mandarin juice have characteristic distribution in the model space, and that the two types of juice can be distinguished by PCA of their VOC concentrations. Figure 3A shows that the Hotelling’s T2 test had only a few outliers, far fewer than the number of samples, and none of them appeared in the large error area (Hot T2 > 11.32, F Res > 13.75). Moreover, in Figure 3B, the contributions of the four groups of principal components to the calibration and validation variances were 99.3% and 98.9%, respectively. The calibration variance curve was largely consistent with the validation variance curve. The above results show that this model can reflect the characteristics of the original sample sufficiently.

### 3.3. Identification of Sweet Orange Juice Mixed With Mandarin Juice

**Figure 4:**
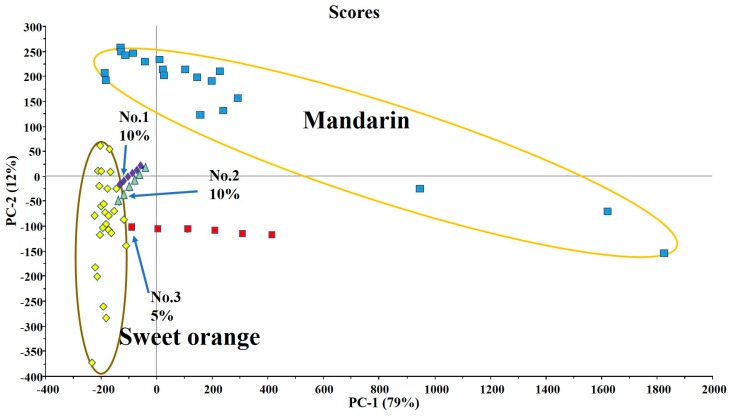
Discrimination of sweet orange juices mixed with different proportions of mandarin juices. No. 1 mixture series are made of Olinda Valencia orange and Xinshengxi No.3 Ponkan juices; No. 2 mixture series are made of Hamlin orange and 2003-4 Satsuma juices; and the No. 3 mixture series are made of Long-leaf orange and Nanfeng mandarin juices. The percentage is the volume fraction of mandarin juices that blended into orange juices. Each group of six points represents a different volume percentage (v: v = 5:95, 10:90, 15:85, 20:80, 25:75, and 30:70).

The obtained discriminant model was used to identify and verify sweet orange juice mixed with mandarin juice. The presented VOCs and their contents in the mixed juices were identified according to the experimental method, and the data of each substance were substituted into the PCA discrimination space for identification. As shown in Figure 4, the points of the mixed juices with low adding proportions were close to the boundary of the sweet orange juice group. With the increase in the proportion of mandarin juice, the sample points of blended orange juice became closer to the mandarin juice group. When the mixing ratio was 10%, samples No. 1 and No. 2 were significantly away from the orange juice group. When the mixing ratio was 5%, sample No. 3 was still beyond the orange juice group. Therefore, blended orange juice can be visually identified with a blending proportion of ≥10%.

In addition, it can be seen that the distance between the respective points of samples No. 1 and No. 2 is significantly shorter than the distance between the points in sample No. 3. In other words, the farther the distance between sweet orange (the yellow points) and mandarin (the blue points) cultivars in the PCA discriminant model, the larger the distance between sample points of the mixed orange juice with different mixing proportions of the combination, and the more obvious the effect. This method suits for determination if sweet orange juice was added with botanically pure mandarin juice, but was not designed to deal with adulteration using orange or mandarin hybrids, such as the Shiranui tangor, Murcott tangerine, Kiyomi tangor, etc. The distribution characteristics of volatile organic compounds of these hybrids may be between the sweet orange and mandarin juices, and the sample scores of hybrid oranges in the above discriminant space may fall between the clusters of sweet orange and mandarin. However, because these hybrids normally have a nice fresh taste, their fresh fruits are sold at a high price, and they are not likely to be used for juicing and blending.

In this study, we collected almost all the major varieties currently used for juicing in sufficient quantities, and established their VOCs database differentiating model. Types and contents of VOCs in the sample citrus only needed to be determined, then the target VOCs input into the identification model, to identify the sample citrus juice. This model used the difference of VOCs between sweet orange juice and mandarin juice to distinguish the two species and quickly identify whether orange juice was mixed with mandarin juice. In China and worldwide markets, mandarin juice is often added to NFC sweet orange juice to reduce costs, and sometimes the product is fraudulently labeled as 100% sweet orange juice. There are no big differences in color and taste between the mixed juice and the pure orange juice. At present, there is no official detection method specifically used to distinguish between sweet orange juice and mandarin juice. The establishment of this method could be an option to solve the adulteration problems.

## 4. Conclusions

The volatile organic compounds in citrus juice were determined by HS-SPME–GC–MS. It was found that 20 and nine characteristic VOCs were identified in sweet orange juice and citrus juice, respectively. Then 32 common VOCs were identified in both species of juices, and their concentrations were different between the two species. By determining whether an orange juice sample contains the characteristic VOCs of mandarin juice, we can preliminarily determine whether the sample was blended with mandarin juice. The identification model of blending sweet orange juice with mandarin juice was established by 32 selected common VOCs, of which concentrations were used in the principal component analysis. The model could visually identify blended orange juice with a 10% or higher volume fraction of mandarin juice.

## Figures and Tables

**Table 1:** Characteristic volatile organic compounds identified in sweet orange juices and mandarin juices.

Juice Type	Compound Category	No.	Calculated RI	Reference RI	Compound Name	CAS Number	Identification
Orange juice	Sesquiterpenes	1	1431	1432	cis-β-Copaene	18252-44-3	MS, RI
		2	1449	1453	α-Guaiene	3691-12-1	MS, RI
		3	1476	1477	γ-Gurjunene	22567-17-5	MS, RI
		4	1499	1499	α-Muurolene	10208-80-7	MS, RI
	Aldehydes	5	1059	1060	2-Octenal	2363-89-5	MS, RI
		6	1159	1160	trans-2-Nonenal	18829-56-6	MS, RI
		7	1408	1408	Decanyl acetate	112-17-4	MS, RI
	Alcohols	8	1138	1138	cis-2,8-p-Menthadien-1-ol	3886-78-0	MS, RI
		9	1150	1144	β-Terpineol	138-87-4	MS, RI
		10	1248	1252	β-Geraniol	106-24-1	MS, RI
		11	1292	1294	p-Mentha-1(7),8(10)-dien-9-ol	29548-13-8	MS, RI
	Esters	12	998	998	Caproic acid ethyl ester	123-66-0	MS, RI
		13	1097	1100	Enanthylic ether	106-30-9	MS, RI
		14	1127	1126	Ethyl 3-hydroxyhexanoate	2305-25-1	MS, RI
		15	1190	1185	Hexyl butanoate	2639-63-6	MS, RI
		16	1308	1312	Nonanol acetate	143-13-5	MS, RI
		17	1408	1408	Decanyl acetate	112-17-4	MS, RI
	Ketones	18	682	680	Ethyl vinyl ketone	1629-58-9	MS, RI
		19	1421	1422	α-Ionone	127-41-3	MS, RI
		20	1807	1814	Nootkanone	4674-50-4	MS, RI
Mandarin juice	Sesquiterpenes	21	1336	1340	δ-Elemene	20307-84-0	MS, RI
		22	1430	1425	γ-Elemene	29873-99-2	MS, RI
		23	1537	1538	α-Cadinene	24406-05-1	MS, RI
		24	1560	1562	Germacrene B	15423-57-1	MS, RI
	Aldehydes	25	1197	1197	trans-4-Decen-1-al	65405-70-1	MS, RI
	Alcohols	26	1153	1150	trans-Isoperitenol	89-79-2	MS, RI
		27	1289	1290	Thyme camphor	89-83-8	MS, RI
	Ketones	28	984	982	Methylheptenone	110-93-0	MS, RI
		29	1256	1253	3-Carvomenthenone	89-81-6	MS, RI

Reference RI: the retention index calculated from the retention time of the volatile organic compounds and n-alkanes on the DB-5MS column. Calculated RI: retention index found in the University of Florida DB-5MS retention index database.

**Table 2:** Common volatile organic compounds identified in sweet orange juices and mandarin juices.

Compound Category	No.	Calculated RI	Reference RI	Name	CAS Number	Identification
Monoterpenes	30	927	928	α-Thujene	2867-05-2	MS, RI
	31	936	936	α-Pinene	80-56-8	MS, RI
	32	953	953	D-Camphene	79-92-5	MS, RI
	33	975	976	Sabenene	3387-41-5	MS, RI
	34	981	981	β-Pinene	127-91-3	MS, RI
	35	990	991	β-Myrcene	123-35-3	MS, RI
	36	1009	1008	α-Phellandrene	99-83-2	MS, RI
	37	1011	1011	δ-3-Carene	13466-78-9	MS, RI
	38	1018	1018	α-Terpinene	99-86-5	MS, RI
	39	1034	1033	d-Limonene	138-86-3	MS, RI
	40	1045	1043	β-trans-Ocimene	3779-61-1	MS, RI
	41	1060	1060	γ-Terpinene	99-85-4	MS, RI
	42	1090	1090	α-Terpinolene	586-62-9	MS, RI
	43	1139	1134	allo-Ocimene	673-84-7	MS, RI
	44	1348	1345	α-Cubebene	17699-14-8	MS, RI
Sesquiterpenes	45	1390	1390	β-Elemen	515-13-9	MS, RI
	46	1440	1441	(+)-Aromadendrene	489-39-4	MS, RI
	47	1451	1457	β-Farnesene	18794-84-8	MS, RI
	48	1457	1455	α-Caryophyllene	6753-98-6	MS, RI
	49	1475	1475	γ-Muurolene	30021-74-0	MS, RI
	50	1482	1487	Germacrene D	23986-74-5	MS, RI
	51	1490	1490	β-Selinene	17066-67-0	MS, RI
	52	1493	1490	Valencene	4630-07-3	MS, RI
	53	1498	1498	α-Selinene	473-13-2	MS, RI
	54	1496	1497	Viridiflorene	21747-46-6	MS, RI
	55	1518	1519	(+)-δ-Cadinene	483-76-1	MS, RI
Aldehydes	56	801	801	Hexanal	66-25-1	MS, RI
	57	853	854	trans-2-Hexenal	6728-26-3	MS, RI
	58	902	903	1-Heptaldehyde	111-71-7	MS, RI
	59	957	957	trans-2-Heptenal	18829-55-5	MS, RI
	60	1004	1002	Octanal	124-13-0	MS, RI
	61	1006	1004	Nonanal	124-19-6	MS, RI
	62	1206	1203	Decanal	112-31-2	MS, RI
	63	1268	1270	α-Citral	141-27-5	MS, RI
	64	1278	1279	Perilla aldehyde	2111-75-3	MS, RI
	65	1307	1306	Undecanal	112-44-7	MS, RI
	66	1409	1409	Dodecanal	112-54-9	MS, RI
Alcohols	67	854	855	Leaf alcohol	928-96-1	MS, RI
	68	867	865	1-Hexanol	111-27-3	MS, RI
	69	1071	1072	1-Octanol	111-87-5	MS, RI
	70	1101	1100	Linalool	78-70-6	MS, RI
	71	1124	1122	cis-p-Menth-2,8-diene-1-ol	7212-40-0	MS, RI
	72	1170	1171	1-Nonanol	143-08-8	MS, RI
	73	1184	1182	4-Terpinenol	562-74-3	MS, RI
	74	1198	1195	α-Terpineol	98-55-5	MS, RI
	75	1220	1217	trans-Carveol	1197-07-5	MS, RI
	76	1225	1224	β-Citronellol	106-22-9	MS, RI
	77	1233	1229	cis-Carveol	1197-06-4	MS, RI
	78	1270	1272	1-Decanol	112-30-1	MS, RI
Esters	79	1195	1193	Caprylic acid ethyl ester	106-32-1	MS, RI
	80	1209	1208	Acetic acid octanyl ester	112-14-1	MS, RI
	81	1285	1285	Bornyl acetic ester	76-49-3	MS, RI
	82	1331	1337	trans-Carvyl acetate	1134-95-8	MS, RI
	83	1346	1350	Terpinyl acetate	80-26-2	MS, RI
	84	1347	1354	Cephrol acetate	150-84-5	MS, RI
	85	1356	1362	Acetic acid neryl ester	141-12-8	MS, RI
	86	1376	1382	Geranyl acetate	105-87-3	MS, RI
	87	1393	1394	Capric acid ethyl ester	110-38-3	MS, RI
Ketones	88	1201	1201	Dihydrocarvone	5948-04-9	MS, RI
	89	1245	1249	L-Carvone	6485-40-1	MS, RI
	90	1445	1448	Geranylacetone	3796-70-1	MS, RI

Reference RI: the retention index calculated from the retention time of the volatile organic compounds and n-alkanes on the DB-5MS column. Calculated RI: retention index found in the University of Florida DB-5MS retention index database. No: continuous numbering following Table 1.

## Supplementary Materials

The following are available online at https://www.mdpi.com/2304-8158/9/4/505/s1, Table S1: Citrus sample information, Table S2: Relative percentage concentration of common monoterpenes volatile substance in sweet orange and mandarin compared to the internal standard express as %, Table S3: Relative percentage concentration of common sesquiterpenes volatile substance in sweet orange and mandarin compared to the internal standard express as %, Table S4: Relative percentage concentration of common alcohols volatile substance in sweet orange and mandarin compared to the internal standard express as %, Table S5: Relative percentage concentration of common aldehydes and ketone volatile substance in sweet orange and mandarin compared to the internal standard express as %.
