# Supplementary material for: Volatile Organic Compounds Profiles to Determine Authenticity of Sweet Orange Juice Using Head Space Gas Chromatography Coupled with Multivariate Analysis
Source: Foods. 2020 Apr 16;9(4):505. doi: 10.3390/foods9040505 (PMC7231238; doi:10.3390/foods9040505)
Supplement: Supplementary file 1 [file foods-09-00505-s001.pdf]

Supplementary table 1. Citrus sample information

Species	Sample number	Local name	English name	Producing area	Mature type	Maturation months
	1	Deltaxiacheng	Delta Valencia Orange	Beibei, Chongqing	late-maturing	late March of the following year
	2	Aerjiliyaxiacheng	Algerian Orange	Xinli, Chongqing	late-maturing	late March of the following year
	3	Aerjiliyaxiacheng	Algerian Orange	Zhongxian, Chongqing	late-maturing	late march of the following year
	4	Aolindaxiacheng	Olinda Valencia Orange	Tu jing, Chongqing	late-maturing	late April of the following year
	5	Aolindaxiacheng	Olinda Valencia Orange	Beibei, Chongqing	late-maturing	late April of the following year
	6	Chuncheng	-	Kaixian, Chongqing	late-maturing	mid-March of the following year
	7	Fulingxiacheng	Valencia Orange	Procurement in Chongqing	late-maturing	mid-May of the following year
	8	Fulingxiacheng	Valencia Orange	Beibei, Chongqing	late-maturing	mid-May of the following year
	9	Hamulintiancheng	Hamlin Orange	Zhongxian, Chongqing	early-maturing	mid-November
	10	Hongxiacheng	Rohde Red Valencia Orange	Beibei, Chongqing	late-maturing	late March of the following year
	11	Jiangan No.35 Xiacheng	-	Beibei, Chongqing	late-maturing	late March of the following year
	12	Jincheng	-	Zhongxian, Chongqing	mid- maturing	late-November
	13	Jincheng	-	Beibei, Chongqing	late-maturing	mid-December
Sweet Orange	14	Tongshui 72-1 Jincheng	-	Zhongxian, Chongqing	mid-maturing	mid-December
	15	Wuyuehong	-	Beibei, Chongqing	late-maturing	late April of the following year
	16	Hongyuxuecheng	Ruby Blood Orange	Beibei, Chongqing	late-maturing	February of the following year
	17	Yuzaocheng	-	Zhongxian, Chongqing	early-maturing	mid-November
	18	Zaojin	-	Zhongxian, Chongqing	early-maturing	mid-October
	19	Changyexiangcheng	-	Beibei, Chongqing	early-maturing	early-November
	20	Changyexiangcheng	-	Jiangjin, Chongqing	late-maturing	late-October
	21	Changyexiangcheng	-	Jiangjin, Chongqing	mid-maturing	late-November
	22	Changyexiangcheng	-	Zhongxian, Chongqing	late-maturing	early-February
	23	Changyexiangcheng	-	Zhongxian, Chongqing	mid-maturing	mid-December

	24	Changyexiangcheng	-	Zhongxian, Chongqing	early-maturing	late-November
	25	Changyexiangcheng	-	Xinli, Chongqing	late-maturing	early-February
	26	Changyexiangcheng	-	Xinli, Chongqing	early-maturing	late-November
	27	Yunguicheng	-	Zhongxian, Chongqing	early-maturing	early-November
Mandarin	28	26-1	-	Beibei, Chongqing	-	early-November
	29	2003-4 Wenzhoumigan	-	Beibei, Chongqing	-	late-November
	30	Aijiju	-	Beibei, Chongqing	-	mid-December
	31	Bayueju	-	Beibei, Chongqing	-	late-November
	32	Caoju	-	Beibei, Chongqing	-	early-December
	33	Dafen No.1 Wenzhoumigan	-	Beibei, Chongqing	especial-early-maturing	early-November
	34	Nanfengmiju	-	Beibei, Chongqing	early-maturing	early-November
	35	Ninghong 73-19 Wenzhoumigan	-	Beibei, Chongqing	mid-maturing	mid-November
	36	Shagan	-	Beibei, Chongqing	mid-maturing	late-November
	37	Taotailangwenzhoumigan	-	Beibei, Chongqing	mid-maturing	mid-November
	38	Tuju	-	Beibei, Chongqing	-	mid-November
	39	Wangcangzhoupigan	-	Beibei, Chongqing	-	late-December
	40	Wugandaju	-	Beibei, Chongqing	-	late January of the following year
	41	Xinshengxi No.3 Ponkan	-	Beibei, Chongqing	-	late-November
	42	Xingyidahongpao	-	Beibei, Chongqing	-	mid-November
	43	Yongshunbingtangju	-	Beibei, Chongqing	-	mid-November
	44	Yuanjiangjiangan	-	Beibei, Chongqing	-	mid-November
	45	Yuanhongxianggan	-	Beibei, Chongqing	-	late-November
	46	Zhoupigan	-	Beibei, Chongqing	-	late-December

Supplementary table 2. Concentration of common monoterpenoid volatile substances in sweet orange and mandarin samples stages express as μg/mL.

Sample number[a]	α-Thujene	α-Pinene	D-Camphene	Sabenene	β-Pinene	β-Myrcene	α-Phellandrene	α-Terpinene	β-trans-Ocimene	γ-Terpinene	α-Terpinolene
1	1.007±0.085	76.857±0.020	ND	0.727±0.043	1.120±0.007	37.009±3.108	0.915±0.063	2.451±0.149	1.795±0.100	8.309±0.012	3.586±0.185
2	0.763±0.010	32.936±1.662	0.425±0.027	2.548±0.299	9.383±0.624	274.752±4.536	8.590±0.857	2.714±0.563	4.591±0.031	14.208±0.056	14.127±2.515
3	1.975±0.078	51.854±1.445	0.422±0.102	7.106±0.835	11.771±1.654	480.489±1.536	13.522±0.098	1.765±0.335	ND	31.866±0.393	29.497±0.343
4	3.987±4.247	27.559±2.439	0.425±0.014	0.808±0.050	ND	176.615±5.352	8.931±0.329	3.119±0.297	4.235±0.665	17.663±0.821	19.511±0.289
5	0.817±0.111	31.657±1.390	0.202±0.001	2.156±0.245	4.676±0.191	245.992±7.230	13.349±0.979	3.207±0.023	6.308±0.198	14.131±0.187	13.693±0.013
6	ND	43.926±0.003	0.281±0.028	ND	ND	317.937±16.469	13.302±2.250	ND	6.496±0.365	13.347±0.335	20.606±1.801
7	ND	19.751±1.307	ND	0.949±0.034	ND	168.764±2.911	ND	ND	2.589±0.270	6.040±0.354	11.617±1.146
8	ND	30.199±0.324	ND	1.646±0.068	ND	238.178±13.765	13.871±0.967	4.275±0.647	7.270±0.555	13.465±0.618	13.705±0.813
9	ND	42.975±1.430	0.293±0.015	ND	5.900±0.321	378.426±5.012	ND	ND	ND	10.814±0.214	19.188±0.329
10	2.758±0.205	38.570±1.699	0.294±0.014	15.429±0.125	2.393±0.056	270.741±23.918	1.591±0.043	2.164±0.114	ND	32.333±2.165	20.903±0.159
11	1.044±0.035	22.298±0.416	ND	6.044±0.735	5.809±0.045	239.806±4.381	3.397±0.217	3.373±0.129	6.626±0.057	8.861±0.173	12.098±0.314
12	1.805±0.079	58.487±0.936	0.341±0.040	6.008±0.267	7.893±0.025	477.412±9.233	0.510±0.010	ND	ND	18.932±1.785	23.126±0.275
13	2.676±0.140	19.622±0.553	ND	10.745±0.748	ND	153.475±10.446	ND	2.016±1.817	2.601±0.223	6.962±0.143	11.565±0.800
14	ND	30.990±0.225	0.583±0.008	ND	ND	259.162±2.615	11.119±0.280	3.062±0.225	3.231±0.024	13.722±0.302	19.164±1.424
15	ND	25.180±0.557	ND	ND	4.360±0.001	179.520±6.762	8.895±0.408	0.350±0.007	2.369±0.312	7.248±0.131	9.553±0.830
16	22.168±1.128	13.628±1.064	ND	52.182±0.851	5.474±0.409	80.123±7.440	7.833±0.062	20.704±1.879	1.766±0.289	35.823±3.557	14.796±1.813
17	2.847±0.232	32.474±1.205	0.309±0.007	14.850±0.256	6.297±0.330	293.383±6.147	ND	3.927±0.223	3.709±0.215	8.128±0.295	14.751±0.070
18	0.791±0.028	44.894±0.216	ND	2.283±0.190	6.278±0.350	247.308±13.766	9.955±0.254	0.352±0.004	2.759±0.199	9.357±0.435	15.336±0.570
19	0.955±0.074	23.851±0.038	0.196±0.009	3.478±0.221	5.127±0.038	230.671±1.742	ND	5.407±0.029	3.263±0.130	13.898±1.431	20.038±0.810
20	1.215±0.049	34.086±2.076	ND	2.503±0.231	5.881±0.580	270.649±7.896	8.760±0.096	3.444±0.147	5.612±0.144	14.181±0.554	16.141±0.503
21	1.425±0.076	55.927±0.415	ND	6.421±0.220	4.745±0.047	302.928±6.097	8.396±0.153	1.240±0.166	ND	17.361±0.508	20.515±1.039
22	5.166±0.172	44.839±2.128	0.299±0.020	19.584±0.581	6.953±0.146	374.461±9.633	6.182±0.001	0.200±0.004	ND	18.112±0.699	22.183±1.440
23	ND	17.709±0.629	ND	ND	ND	138.529±4.657	5.532±0.502	1.688±0.237	1.858±0.148	10.79±0.082	11.034±0.096

24	ND	44.707±2.152	0.241±0.019	0.698±0.005	5.268±0.171	373.426±16.983	13.465±0.201	ND	ND	10.737±0.090	18.910±0.350
25	4.628±0.195	44.014±1.152	ND	19.849±0.754	ND	332.739±8.801	10.439±0.292	ND	ND	16.699±0.549	22.030±0.064
26	7.989±0.059	30.942±0.866	0.501±0.005	23.206±1.198	10.125±0.195	237.693±7.463	19.152±0.378	8.298±0.045	5.606±0.352	16.768±1.475	23.210±1.156
27	0.914±0.012	50.222±1.766	0.434±0.008	2.868±0.086	6.225±0.086	425.164±9.187	ND	ND	ND	16.381±0.233	22.288±3.472
28	0.790±0.094	16.147±0.250	ND	1.109±0.003	2.658±0.006	149.882±3.874	4.708±0.044	6.351±0.121	37.538±1.593	24.426±1.430	12.931±1.117
29	4.698±0.245	34.258±0.201	0.401±0.019	ND	23.111±1.856	169.663±5.311	8.83±0.750	11.838±0.887	8.572±0.292	357.446±9.895	32.828±1.705
30	29.188±0.901	61.270±1.116	8.330±9.699	1.849±0.081	65.756±0.995	98.335±2.815	10.037±0.793	59.891±0.785	3.035±0.329	1095.628±0.009	104.458±0.963
31	2.536±0.093	10.818±0.655	ND	ND	6.852±0.054	37.791±0.619	2.806±0.126	6.007±0.281	1.472±0.004	128.655±1.338	13.137±1.259
32	3.435±0.177	30.222±1.278	ND	ND	4.638±0.102	236.856±13.856	9.025±0.399	7.898±0.003	10.735±0.379	24.794±0.528	15.077±0.959
33	3.571±0.267	12.931±0.263	ND	ND	7.786±0.190	31.271±2.550	1.047±0.044	4.377±0.382	0.941±0.005	106.161±6.965	9.860±0.024
34	7.514±0.261	32.221±0.570	0.744±0.052	ND	39.387±0.789	111.476±0.594	6.961±0.508	18.908±0.838	5.895±0.046	425.285±22.072	43.205±0.655
35	3.391±0.100	20.908±0.320	0.389±0.023	ND	11.375±0.753	43.174±4.455	7.645±0.435	8.709±0.326	1.143±0.073	175.503±2.546	13.108±0.773
36	10.93±0.037	ND	ND	0.602±0.040	0.908±0.090	105.053±2.349	5.064±0.177	2.15±0.030	13.991±0.223	18.313±0.084	6.672±0.214
37	1.503±0.070	8.191±0.623	ND	ND	4.209±0.370	22.932±0.607	1.201±0.132	3.849±0.003	0.915±0.010	92.020±2.647	9.310±0.908
38	5.104±0.236	19.106±0.322	0.264±0.017	0.569±0.006	15.381±0.914	102.297±3.309	5.896±0.180	13.352±0.709	11.535±0.348	312.898±11.575	31.783±0.714
39	9.321±0.004	38.558±0.527	0.486±0.028	0.858±0.038	27.875±0.464	189.681±9.394	9.489±0.456	22.11±1.234	21.640±0.765	486.199±9.868	52.485±1.104
40	6.207±0.101	26.136±1.528	0.318±0.017	1.053±0.136	16.774±1.731	121.143±9.071	6.428±0.343	15.125±0.820	5.938±0.216	346.318±4.323	36.559±0.984
41	66.050±1.209	176.455±8.969	3.395±0.171	56.851±1.211	160.065±3.730	679.924±38.740	ND	47.854±2.103	ND	1938.595±78.693	261.947±8.894
42	6.120±0.119	25.043±0.662	0.278±0.020	0.723±0.084	13.652±0.127	105.230±4.270	5.786±0.177	10.692±0.047	5.535±0.051	230.481±8.698	24.837±1.801
43	1.429±0.086	8.687±0.378	ND	ND	5.156±0.276	36.594±2.912	2.591±0.251	4.422±0.322	2.395±0.194	95.699±0.798	8.739±0.289
44	7.475±0.171	28.085±0.370	0.343±0.004	0.735±0.066	19.735±1.872	138.981±7.001	6.978±0.081	15.493±0.432	17.397±0.244	401.027±9.780	44.163±0.920
45	0.502±0.007	10.771±1.485	ND	ND	2.009±0.057	78.749±1.412	4.380±0.216	8.302±0.118	3.498±0.115	21.806±1.891	7.289±0.348
46	34.314±1.239	168.330±57.598	1.710±0.020	5.201±0.187	113.224±15.739	553.008±19.241	9.658±11.228	8.215±0.120	ND	1758.580±212.607	265.223±4.320

[a]: The sample numbers in the supplementary table 2 corresponds to those in supplementary table 1

Note: the results were shown as mean ± standard deviation with 3 decimal places and ND= not detected.

Supplementary table 3. Concentration of common sesquiterpenoids volatile substance in sweet orange and mandarin stages express as μg/mL.

Sample number[a]	α-Caryophyllene	γ-Muurolene	β-Selinene	Valencen	α-Selinine	Viridiflorene	(+)-δ-Cadinene
1	1.694±0.199	ND	4.536±0.007	55.810±0.008	5.473±0.098	1.485±0.397	0.943±0.045
2	1.646±0.361	ND	8.046±0.214	100.100±5.391	9.648±0.059	2.177±0.109	9.950±1.512
3	2.152±0.083	ND	10.133±0.197	116.969±7.597	12.721±1.017	4.261±0.083	11.045±0.468
4	2.675±0.141	ND	34.305±2.220	428.960±13.028	31.231±2.573	8.934±0.480	3.096±0.322
5	1.558±0.191	ND	23.697±1.256	274.004±20.200	25.285±0.866	5.291±0.162	3.799±0.115
6	4.569±0.474	ND	12.041±1.534	185.580±13.403	16.318±0.291	2.970±0.063	23.052±1.297
7	1.379±0.102	ND	12.692±0.968	193.971±9.716	15.084±0.583	3.600±0.313	3.943±0.114
8	2.392±0.135	ND	32.799±2.307	413.370±14.814	36.796±1.053	7.983±0.320	5.016±0.165
9	2.402±0.083	ND	3.440±0.434	61.656±0.628	5.588±0.165	0.567±0.801	3.052±4.317
10	2.091±0.088	ND	19.523±1.570	262.374±10.901	20.521±1.321	4.309±0.46	6.384±0.172
11	1.200±0.061	ND	18.094±0.599	223.145±10.182	18.439±0.749	4.140±0.544	3.543±0.342
12	2.761±0.058	2.387±0.022	3.761±0.093	57.930±2.018	7.674±0.326	1.035±0.008	14.707±0.345
14	10.671±12.784	ND	9.783±0.341	160.242±5.267	10.991±0.790	2.430±0.168	6.599±0.410
13	1.436±0.014	7.861±0.006	26.855±0.453	321.106±8.280	27.439±0.923	7.631±0.325	2.335±0.044
15	2.225±0.221	2.404±0.063	21.762±2.359	230.535±14.617	20.264±0.610	3.865±0.034	2.892±0.305
16	0.873±0.082	7.212±0.179	19.133±1.001	269.452±16.509	19.366±0.942	5.056±0.680	9.796±11.434
17	0.904±0.050	3.977±0.214	10.326±0.732	206.815±7.561	10.407±0.891	2.406±0.298	3.427±0.208
18	1.338±0.092	ND	14.152±1.164	214.261±11.456	13.913±1.283	3.686±0.056	4.150±0.342
19	1.558±0.105	ND	22.082±2.588	305.226±14.006	21.498±2.688	6.200±0.242	3.451±0.162
20	1.540±0.161	ND	14.532±1.372	235.750±14.778	15.504±0.984	3.658±0.037	5.052±0.256
21	2.136±0.002	ND	17.117±0.827	232.869±7.534	16.418±0.859	3.475±0.239	7.639±0.547
22	3.015±0.243	ND	35.150±1.781	403.749±9.364	36.612±3.517	7.326±0.601	9.158±0.216
23	0.652±0.010	ND	4.548±0.020	75.690±2.008	4.891±0.091	ND	1.838±0.313
24	2.670±0.051	2.476±0.082	8.144±0.116	123.964±3.538	10.700±0.609	2.104±0.233	10.552±0.269
25	2.911±0.340	ND	36.597±3.075	403.688±12.118	36.186±0.695	8.798±0.811	10.478±0.800
26	4.062±0.001	ND	57.787±0.253	631.866±8.446	53.982±1.155	11.755±1.732	10.497±0.066
27	1.697±0.036	ND	7.334±0.191	15.441±4.864	8.472±0.253	1.909±0.417	5.936±0.188
28	3.386±0.151	0.970±0.026	0.839±0.029	6.649±0.543	2.659±0.023	ND	6.697±0.065
29	0.975±0.066	1.231±0.129	ND	64.641±5.361	6.883±0.201	1.526±0.155	3.258±0.085
30	0.742±0.060	ND	ND	0.219±0.009	1.168±0.049	2.010±0.127	0.958±0.051
31	2.627±0.069	0.788±0.099	0.952±0.016	6.379±0.247	1.914±0.017	1.383±0.113	3.756±0.011
32	6.632±0.286	1.619±0.061	3.277±0.088	ND	3.225±1.560	5.718±0.052	11.425±0.286
33	0.993±0.086	ND	0.575±0.008	14.183±0.212	1.685±0.022	0.824±0.011	0.994±0.073
34	1.383±0.014	0.355±0.020	ND	0.311±0.001	1.179±0.027	16.266±0.070	2.157±0.017
35	3.238±0.011	1.874±0.103	2.341±0.045	18.789±1.612	3.974±0.141	5.533±0.155	5.160±0.455
36	1.232±0.083	0.531±0.005	0.621±0.004	7.560±0.054	2.077±0.020	1.674±0.151	3.130±0.124
37	ND	1.436±0.091	1.141±0.089	6.697±0.367	2.240±0.028	1.074±0.052	2.389±0.108

38	0.824±0.005	ND	0.490±0.023	ND	0.735±0.047	0.810±0.122	2.530±0.063
39	1.669±0.053	1.227±0.143	0.525±0.012	4.711±0.118	1.921±0.022	ND	4.133±0.464
40	0.904±0.062	0.134±0.003	0.342±0.038	1.149±0.038	0.715±0.072	ND	1.303±0.046
41	4.389±0.249	0.996±0.021	1.284±0.064	4.866±0.224	3.361±0.304	2.494±0.112	6.287±0.297
42	1.051±0.077	ND	0.184±0.007	0.284±0.011	0.452±0.065	ND	0.884±0.024
43	0.209±0.296	ND	ND	ND	ND	ND	0.658±0.004
44	1.368±0.070	0.411±0.006	0.754±0.035	ND	1.081±0.095	2.909±0.320	4.238±0.113
45	0.643±0.095	ND	0.336±0.004	0.901±0.027	0.545±0.034	0.600±0.055	0.847±0.082
46	12.48±0.392	5.688±0.378	4.494±0.322	8.281±0.095	15.057±1.175	ND	27.852±2.047

[a]: The sample serial number in the supplementary table 3 corresponds to that in supplementary table 1.

Note: the results were shown as mean ± standard deviation with 3 decimal places and ND= not detected.

Supplementary table 4. Concentration of common alcohols volatile substance in sweet orange and mandarin stages express as μg/mL.

Sample number[a]	Leaf alcohol	1-Octanol	Linalool	1-Nonanol	4-Terpinenol	α-Terpineol	β-Citronellol	1-Decanol
1	ND	5.542±0.040	49.885±0.315	ND	7.469±0.317	3.669±0.255	3.621±0.209	0.461±0.007
2	7.397±0.229	39.672±4.441	136.865±9.744	4.988±0.224	30.565±2.488	15.307±1.417	20.066±0.497	11.640±1.123
3	1.897±0.070	49.003±2.478	153.013±3.318	6.056±0.109	52.138±2.932	13.240±0.355	35.253±0.310	15.095±0.717
4	8.596±0.209	17.786±0.252	106.264±0.392	2.918±0.015	45.019±1.994	9.047±0.147	7.724±8.353	3.602±0.122
5	2.792±0.216	18.130±0.754	54.307±2.897	1.566±0.017	22.022±2.038	21.853±1.263	11.550±0.468	3.078±0.434
6	12.243±0.288	5.145±0.207	59.285±3.175	ND	36.525±2.130	7.856±0.413	5.250±0.214	ND
7	ND	4.009±0.040	71.020±2.345	ND	7.983±0.305	6.113±0.187	7.088±0.010	ND
8	2.575±0.165	7.957±0.001	22.067±1.368	ND	23.662±0.606	13.702±0.068	3.196±0.066	1.764±0.061
9	9.309±0.477	18.160±2.059	88.143±3.619	2.557±0.177	22.760±0.947	11.338±0.834	6.780±0.101	2.654±0.258
10	4.563±0.027	37.343±0.618	120.966±3.531	4.258±0.347	29.769±1.892	10.060±0.819	27.417±0.258	13.089±0.354
11	10.217±0.015	14.164±0.111	87.618±1.679	2.109±0.297	11.596±0.761	6.547±0.027	17.204±1.373	4.681±0.285
12	8.603±0.595	12.833±0.639	104.753±0.999	1.255±0.120	36.169±0.076	10.298±0.008	8.673±0.020	2.016±0.135
14	21.549±0.138	33.788±1.633	106.217±8.745	4.249±0.112	40.852±3.083	17.902±1.749	9.455±0.209	3.642±0.071
13	13.185±0.017	15.014±0.934	106.156±5.204	1.668±0.256	4.002±0.009	6.412±0.021	11.873±0.276	2.689±0.098
15	3.495±0.168	1.014±0.001	1.235±0.031	ND	7.950±0.344	2.104±0.107	1.129±0.020	ND
16	9.790±0.487	3.697±0.053	17.434±1.190	ND	30.569±1.295	2.596±0.088	ND	ND
17	5.507±0.497	7.624±0.356	21.437±1.985	ND	10.571±0.209	5.025±0.206	7.777±0.126	0.861±0.053
18	10.121±0.044	4.131±0.108	44.289±0.090	ND	17.564±0.740	3.875±0.075	5.526±0.069	ND
19	3.733±0.056	6.612±0.038	116.996±1.327	ND	18.081±0.702	6.569±0.281	13.288±0.120	1.202±0.118
20	4.271±0.160	10.433±0.754	229.354±14.357	ND	28.059±1.267	10.249±0.587	8.842±0.047	2.205±0.036
21	14.184±0.604	9.365±0.091	74.772±0.752	2.204±0.103	30.259±0.999	6.720±0.129	6.080±0.119	2.497±0.518
22	1.552±0.175	9.897±0.303	32.995±1.235	1.587±0.078	20.017±0.449	3.751±0.214	5.933±0.733	2.216±0.052
23	15.001±0.286	10.350±0.223	68.716±2.470	ND	22.625±0.602	6.314±0.041	7.853±0.145	0.373±0.527
24	11.493±0.004	13.833±0.71	62.257±0.457	1.691±0.180	26.539±0.440	10.188±0.316	6.320±0.036	1.979±0.228
25	8.898±0.106	13.58±0.132	62.229±1.597	1.431±0.078	22.148±0.038	6.181±0.680	10.210±0.106	4.040±0.154
26	12.379±0.499	6.740±0.340	31.644±0.835	2.402±0.280	20.161±0.999	4.619±0.216	5.426±0.203	2.395±0.289
27	10.639±0.378	17.670±1.048	19.602±1.109	2.347±0.023	4.127±0.041	11.874±0.288	10.946±0.143	3.047±1.012

28	2.607±0.192	4.636±6.557	118.834±6.268	2.140±0.085	23.266±0.195	9.106±0.670	4.530±0.204	6.913±0.363
29	9.449±0.646	6.937±0.524	7.997±0.062	0.941±0.024	11.495±0.208	9.339±0.369	4.223±0.241	1.092±0.027
30	ND	1.727±0.185	12.712±0.539	ND	31.373±0.700	21.701±0.100	45.804±0.937	0.661±0.061
31	4.389±0.232	1.532±0.017	19.797±0.639	0.433±0.042	2.404±0.057	3.855±0.110	ND	ND
32	1.934±0.059	14.187±1.376	31.703±0.479	ND	13.170±0.075	8.621±0.516	28.884±0.650	8.699±0.002
33	3.181±0.054	ND	11.760±0.172	ND	2.042±0.006	1.804±0.039	ND	ND
34	0.519±0.020	9.104±0.125	29.604±0.326	1.452±0.005	17.172±0.610	128.898±4.823	ND	2.337±0.101
35	13.507±0.464	2.816±0.301	9.197±0.106	0.890±0.032	5.297±0.047	7.501±0.019	ND	ND
36	7.016±0.003	18.051±1.060	106.161±3.156	6.095±0.115	5.195±0.261	14.983±0.033	11.246±0.385	12.047±0.744
37	3.063±0.210	1.537±0.023	4.647±0.035	ND	2.635±0.116	3.399±0.156	ND	ND
38	2.765±0.133	6.751±0.350	52.528±0.587	1.377±0.117	11.478±0.112	13.159±0.149	12.234±0.457	2.184±0.329
39	1.325±0.079	11.372±0.017	32.648±0.075	ND	11.948±0.092	12.689±0.157	12.729±0.216	2.199±0.104
40	ND	5.721±0.339	60.174±0.115	1.709±0.088	14.346±0.149	7.533±0.128	6.640±0.557	1.773±0.039
41	ND	ND	170.939±4.726	5.965±0.111	88.543±1.881	25.645±0.605	47.333±0.612	19.116±1.135
42	ND	5.629±0.339	110.863±2.336	0.805±0.020	7.273±0.473	9.309±0.278	6.680±0.485	1.151±0.065
43	1.698±0.045	3.716±0.363	94.978±5.454	0.794±0.001	5.640±0.332	9.789±0.542	2.815±0.032	0.820±0.049
44	0.655±0.005	4.044±0.132	15.498±0.617	1.007±0.090	9.770±0.336	13.170±0.450	6.117±0.328	1.862±0.218
45	4.802±0.272	21.510±1.129	241.802±12.808	0.543±0.033	22.393±0.659	16.796±0.187	18.557±0.600	4.752±0.053
46	ND	ND	53.291±4.174	1.211±0.084	28.614±1.246	25.590±1.273	31.222±2.974	7.928±0.045

[a]: The sample serial number in the supplementary table 4 corresponds to that in supplementary table 1.

Note: the results were shown as mean ± standard deviation with 3 decimal places and ND= not detected.

Supplementary table 5. Concentration of common aldehydes and ketones volatile substance in sweet orange and mandarin stages express as μg/mL.

Sample number[a]	Octanal	Nonanal	Decanal	Perilla aldehyde	L-Carvone	Geranylacetone
1	2.541±0.096	2.440±0.256	1.035±0.136	1.300±0.049	7.443±0.102	5.580±0.008
2	2.719±0.153	14.640±2.272	6.828±0.405	5.423±0.160	40.281±3.010	11.583±0.078
3	ND	7.565±0.219	3.897±0.112	4.268±0.600	17.674±0.211	ND
4	10.200±0.633	6.570±9.292	6.061±0.251	3.970±0.132	34.048±0.069	5.237±0.159
5	ND	4.427±0.066	2.266±0.267	1.383±0.158	9.588±0.772	ND
6	5.092±0.011	4.733±0.771	2.260±0.151	11.154±0.499	18.355±0.797	4.592±0.036
7	21.594±1.212	12.856±0.202	25.182±0.314	4.152±0.166	18.791±0.254	2.964±0.108
8	ND	3.263±0.152	1.901±0.111	1.470±0.042	7.964±0.051	2.537±0.004
9	4.196±0.200	4.648±0.532	1.753±0.237	5.862±0.246	23.865±0.160	3.503±0.063
10	7.135±0.057	4.308±0.411	8.310±0.781	5.184±0.391	4.260±0.022	12.119±0.062
11	4.707±0.298	4.055±0.471	2.905±0.109	2.034±0.107	8.379±0.185	11.018±0.185
12	4.989±0.111	4.628±0.175	3.147±0.055	14.663±0.433	22.026±0.442	3.130±0.017
14	ND	3.588±0.226	4.479±0.033	4.333±0.249	38.008±1.542	ND
13	4.456±0.105	3.988±0.523	2.081±0.203	3.002±0.042	5.467±0.384	ND
15	2.131±0.258	5.768±0.153	1.734±0.083	ND	2.107±0.270	2.900±0.353
16	4.320±0.049	5.116±0.400	2.365±0.237	ND	3.432±0.201	ND
17	2.697±0.149	2.759±0.136	2.775±0.252	2.325±0.151	6.408±0.374	4.181±0.052

18	2.993±0.067	5.187±0.146	2.071±0.004	11.003±0.216	25.634±2.633	ND
19	3.467±0.367	5.612±0.581	3.020±0.032	1.756±0.084	13.995±0.532	6.426±0.127
20	9.802±0.105	7.350±0.561	7.696±0.211	5.364±0.399	38.205±2.644	6.037±0.062
21	8.223±0.011	6.616±0.268	3.819±0.293	17.329±0.072	30.496±0.343	5.382±0.150
22	ND	4.717±0.031	2.604±0.022	3.605±0.191	11.654±0.236	11.612±0.260
23	6.428±0.433	4.762±0.103	1.355±0.144	8.998±0.112	18.808±0.487	1.456±0.091
24	3.794±0.149	8.213±0.029	4.769±0.024	8.098±0.507	36.291±2.110	6.537±0.072
25	9.454±0.006	14.651±1.386	5.877±0.150	8.078±0.667	55.090±0.871	19.185±0.736
26	10.904±0.969	16.885±0.196	3.025±0.203	5.684±0.306	42.376±0.729	16.421±0.058
27	ND	4.274±0.243	2.201±0.041	9.579±0.070	31.650±1.640	ND
28	4.352±0.250	8.938±0.063	7.469±0.069	2.145±0.101	24.462±0.25	20.708±0.380
29	2.504±0.193	5.674±0.093	3.445±0.099	0.779±0.047	2.972±0.172	ND
30	2.851±0.033	3.325±0.174	3.614±0.025	1.362±0.114	0.790±0.021	ND
31	1.757±0.097	4.871±0.697	2.846±0.012	ND	ND	2.692±0.001
32	3.374±0.170	5.322±0.049	5.612±0.027	2.199±0.230	4.300±0.013	ND
33	1.742±0.118	5.016±0.485	0.781±0.054	ND	ND	ND
34	0.919±0.001	4.280±0.067	3.218±0.224	ND	0.701±0.117	ND
35	7.514±0.300	29.731±0.076	2.767±0.182	ND	ND	3.688±0.068
36	7.901±0.380	8.050±0.042	12.070±0.258	2.213±0.048	2.298±0.071	4.553±0.209
37	3.638±0.146	5.487±0.147	1.160±0.089	ND	0.410±0.001	ND
38	2.896±0.044	5.491±0.248	2.462±0.115	1.672±0.007	1.512±0.005	2.135±0.126
39	2.577±0.007	4.225±0.085	1.636±0.115	1.345±0.142	1.656±0.169	2.257±0.038
40	1.132±1.601	6.319±0.087	5.022±0.257	0.863±0.059	1.129±0.007	1.612±0.013
41	ND	12.552±0.616	95.863±2.081	16.684±0.678	4.531±0.197	ND
42	3.836±0.145	3.837±0.054	1.648±0.188	ND	0.776±0.036	1.408±0.153
43	3.948±0.241	7.901±0.166	2.878±0.371	ND	0.443±0.030	1.938±0.017
44	2.610±0.003	3.494±0.057	2.228±0.153	0.461±0.009	1.319±0.121	3.483±0.091
45	4.260±0.029	8.430±0.025	3.031±0.368	2.368±0.168	1.437±0.069	ND
46	ND	9.282±0.404	103.843±12.510	18.852±0.317	6.169±0.491	ND

[a]: The sample serial number in the supplementary table 2 corresponds to that in supplementary table 1.

Note: the results were shown as mean ± standard deviation with 3 decimal places and ND= not detected.
